# Supplementary material for: Wound Fluid from Breast Cancer Patients Undergoing Intraoperative Radiotherapy Exhibits an Altered Cytokine Profile and Impairs Mesenchymal Stromal Cell Function
Source: Cancers (Basel). 2021 Apr 29;13(9):2140. doi: 10.3390/cancers13092140 (PMC8124792; doi:10.3390/cancers13092140)
Supplement: Supplementary file 1 [file cancers-13-02140-s001.zip › cancers-1153921-supplementary.pdf]

cancers

Supplementary Material

Wound Fluid from Breast Cancer Patients Undergoing Intraoperative Radiotherapy Exhibits an Altered Cytokine Profile and Impairs Mesenchymal Stromal Cell Function

Anne Wuhrer, Stefanie Uhlig, Benjamin Tuschy, Sebastian Berlit, Elena Sperk, Karen Bieback and Marc Sütterlin

Table S1: Reagents used for immunophenotyping.

Name / Antigen	Clone	Fluorochrome	Manufacturer
**Myeloid panel**			
CD15 (neutrophils)	HI98	FITC	Becton Dickinson
CD16 (NK cells, phagocytes)	3G8	APC-Cy7	Becton Dickinson
CD45 (leucocytes)	HI30	PE-Cy7	Becton Dickinson
CD14 (mainly monocytes)	MφP9	PerCP-Cy5.5	Becton Dickinson
CD64 (mature myeloid cells)	10.1	PE	Becton Dickinson
CD56 (NK cells)	NCAM16.2	APC	Becton Dickinson
TruCOUNT™ tubes			Becton Dickinson
FcR Blocking Reagent			Miltenyi Biotec
**Lymphoid panel**			
CD3 (t cells)	UCHT1	APC/Cy7	BioLegend
CD4 (t helper cells)	RPA-T4	PE	Becton Dickinson
CD8 (cytotoxic t cells)	RPA-T8	FITC	Becton Dickinson
CD45 (leucocytes)	HI30	PE-Cy7	Becton Dickinson
CD69 (early activation)	FN50	PerCP	BioLegend
CD154 (activation)	TRAP1	APC	Becton Dickinson
TruCOUNT™ tubes			Becton Dickinson
FcR Blocking Reagent			Miltenyi Biotec
**Treg panel**			
CD4	RPA-T4	FITC	Becton Dickinson
CD25	M-A251	APC	Becton Dickinson
CD127	HIL-7R-M21	PE-Cy7	Becton Dickinson
CD196	11A9	PerCP-Cy5.5	Becton Dickinson
FoxP3	259D/C7	PE	Becton Dickinson
**Annexin V/PI staining**			
CD45	HI30	PE-Cy7	Becton Dickinson
Annexin V-APC			Becton Dickinson
Annexin V-pure			Becton Dickinson
FcR Blocking Reagent			Miltenyi Biotec

Table S2. Rating strategies for the immunophenotyping of the myeloid, lymphoid and Treg panel.

**Myeloid panel**	
Monocytes:	CD45+ → CD14+
Neutrophils:	CD45+ → CD15+, CD16+
NK cells:	FSC/SSC (lymphocytes, morphological) → CD45+, CD64+
	subtyping: CD56 vs. CD16
Mature myeloid cells:	CD45+ → CD64+
**Lymphoid panel**	
T cells:	CD45+ → CD3+
T helper cells:	CD45+ → CD3+ → CD4+
Cytotoxic T cells:	CD45+ → CD3+ → CD8+
**Activation markers**	
early:	CD45+ → CD3+ → CD69+
late:	CD45+ → CD3+ → CD154+
**Treg panel**	
FSC/SSC (lymphocytes, morphological)	→ CD4, CD25
	→ CD127, FoxP3
	→ CD196

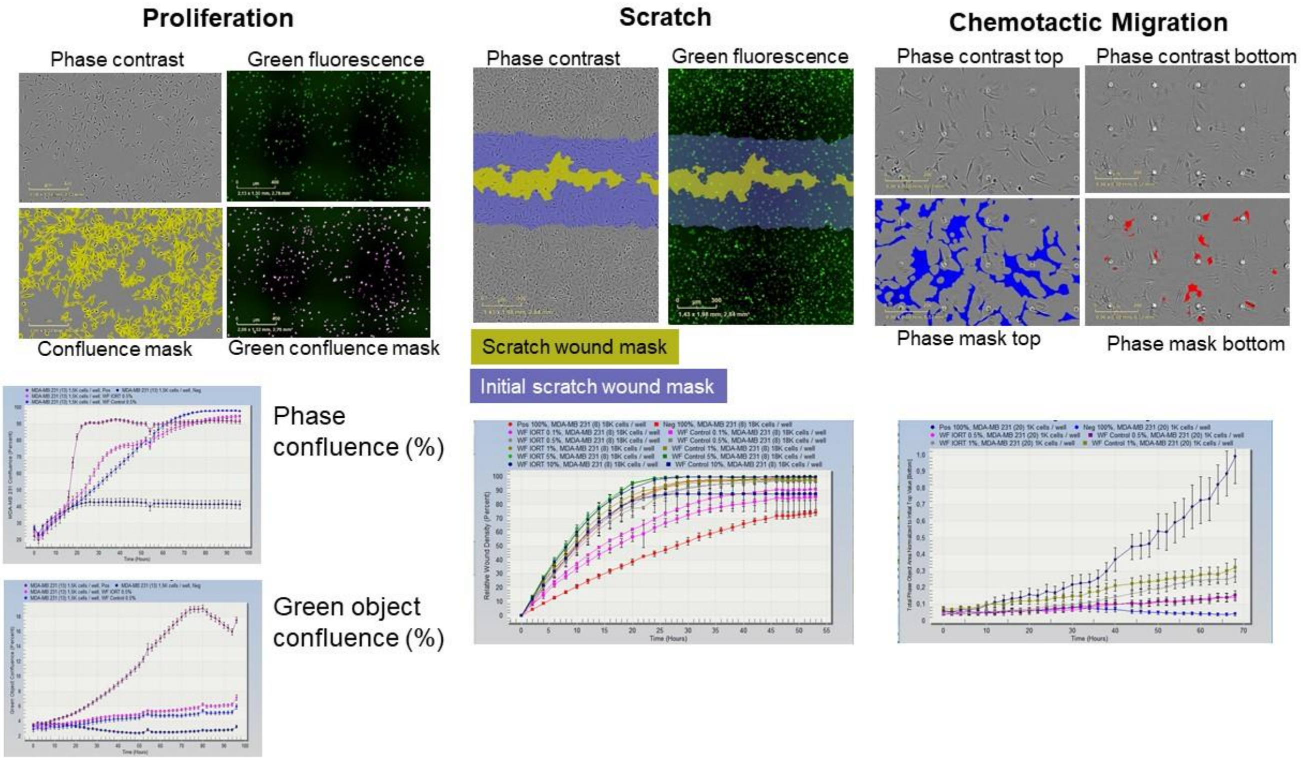

Figure S1. Captures and analysis masks monitored during MDA-MB 231 experiments with IncuCyte ZOOM® that allow the comparison of phase contrast and nuclear GFP confluence. Cell proliferation was quantified as percent confluence using either nuclear GFP values (proliferation) or phase contrast values (scratch wound healing and chemotactic migration).
